# Myeloarchitectonic cortical parcellation data for contemporary neuroimaging – the Vogt-Vogt legacy in the 21st century

**DOI:** 10.1016/j.dib.2023.108999

**Published:** 2023-02-20

**Authors:** Niels Alexander Foit, Seles Yung, Hyo Min Lee, Andrea Bernasconi, Neda Bernasconi, Seok-Jun Hong

**Affiliations:** a Neuroimaging of Epilepsy Laboratory, McConnell Brain Imaging Center, Montreal Neurological Institute, Montreal, QC, Canada; b Department of Neurosurgery, Medical Center - University of Freiburg, Freiburg, Germany; c Center for the Developing Brain, Child Mind Institute, NY, USA; d Center for Neuroscience Imaging Research, Institute for Basic Science, Suwon, Korea; e Department of Biomedical Engineering, Sungkyunkwan University, Suwon, Korea

**Keywords:** Myeloarchitectonics, Cytoarchitectonics, Myelin, MRI, Histology, Atlas

## Abstract

Obtaining precise and detailed parcellations of the human brain has been a major focus of neuroscience research. Here, we present a multimodal dataset, MYATLAS, based on histology-derived myeloarchitectonic parcellations for use with contemporary neuroimaging analyses software. The core of MYATLAS is a novel 3D neocortical, surface-based atlas derived from legacy myeloarchitectonic histology studies. Additionally, we provide digitized quantitative laminar profiles of intracortical myelin content derived from postmortem photometric data, cross-correlated with *in vivo* myeloarchitectonic features obtained by quantitative MRI mapping. Moreover, congregated, digitized and quality-improved Vogt-Vogt legacy histology data is made available. Finally, to allow for cross-modality correlations, maps of quantitative myelin estimates and corresponding von Economo-Koskinas’ cytoarchitectonic features are also included. We share all necessary surface and volume-based registration files as well as shell scripts to facilitate applications of MYATLAS to future *in vivo* MRI studies.

**Specifications Table**

**Table:** 

Subject	Neuroscience - General
Specific subject area	MRI processing, brain segmentation and atlas creation, neuroimaging, myeloarchitectonic data, myelin proxy imaging
Type of data	Raw data Nifti files .label / .annot files .scene files Bash shell scripts
How the data were acquired	Histological data were extracted from digitized copies of original Vogt-Vogt publications as described further below and in the original publication. Digitized histological slices were normalized to ensure homogenous quality for comparisons. Manual, landmark-guided segmentation was used to create the 3D myeloarchitectonic atlas. Utilizing FreeSurfer, quantitative histological myelin contents were correlated with processed *in vivo* T1q MRI data and cytoarchitectonic features, respectively. All raw *in vivo* data originate from the LEMON dataset, of which acquisition protocols and preprocessing steps have been previously described in detail.
Data format	Raw Processed Analyzed
Description of data collection	All data described in this article is secondary data derived from historical (Vogt) and open MRI data (LEMON). Original Vogt-Vogt histology data were included when source files were a) available and b) image quality was sufficient to apply postprocessing steps. This resulted in digitized histology slices (digitized) and light absorption spectrums (newly created). Raw quantitative T1w MRI data was obtained from 202 randomly selected healthy subjects of the LEMON dataset. The sources are described in the following paragraph.
Data source location	1) Raw data on Vogt-Vogt histology are available in print or digitized from the German National Library (Deutsche Nationalbibliothek) on demand or publicly available from thehumanbrain.info website. Other sources have been published online: R. Nieuwenhuys, C.A.J. Broere, L. Cerliani, A new myeloarchitectonic map of the human neocortex based on data from the Vogt–Vogt school, Brain Struct. Funct. 220 (2015) 2551–2573. 10.1007/s00429–014–0806–9. R. Pijnenburg, L.H. Scholtens, D.J. Ardesch, S.C. de Lange, Y. Wei, M.P. van den Heuvel, Myelo- and cytoarchitectonic microstructural and functional human cortical atlases reconstructed in common MRI space, NeuroImage. 239 (2021) 118274. 10.1016/j.neuroimage.2021.118274. C. Vogt, O. Vogt, Allgemeine Ergebnisse unserer Hirnforschung, 1919. (https://d-nb.info/365623059) E.-G. Batsch, Die myeloarchitektonische Untergliederung des Isocortex parietalis beim Menschen, J. Hirnforsch. (1955) 46. (https://www.thehumanbrain.info/brain/db_literature/vogt_o_1911_isocx_parietalis.pdf) E.H. Strasburger, Die myeloarchitektonische Gliederung des Stirnhirns beim Menschen und Schimpansen, (1937) 42. (https://www.thehumanbrain.info/database/db_literature/StrassburgerEduard.pdf) A. Hopf, Über die Verteilung myeloarchitektonischer Merkmale in der Stirnhirnrinde beim Menschen, (1956) 23. (https://www.thehumanbrain.info/database/db_literature_structures/HopfAdolf3.pdf)
	A. Hopf, Über die Verteilung myeloarchitektonischer Merkmale in der Scheitellappenrinde beim Menschen, (1957) 26. (https://thehumanbrain.info/brain/db_literature/HopfAdolf2.pdf) A. Hopf, Über die Verteilung myeloarchitektonischer Merkmale in der isokortikalen Schläfenlappenrinde beim Menschen, (1955) 19. (https://www.thehumanbrain.info/brain/db_literature/HopfAdolf1.pdf) H. Brockhaus, Die Cyto- und Myeloarchitektonik des Cortex claustralis und des Claustrum beim Menschen, J. Fuer Psychol. Neurol. 49 (1940) 100. (https://www.thehumanbrain.info/brain/db_literature/BrockhausCortexclaustralis.pdf) *2) In vivo*MRI data: Leipzig Study for Mind-Body-Emotion Interactions (LEMON) dataset www.openneuro.org/datasets/ds000221/versions/00002
Data accessibility	All data is available for download from both BALSA Neuroimaging Database and the GitHub node, respectively, while original raw data is available from the source. In compliance with NeuroImage review requirements, both data archives are given since BALSA allows for direct data query with the connectome workbench, as mentioned in the original publication.
Related research article	Foit NA, Yung S, Lee HM, Bernasconi A, Bernasconi N, Hong S-J: A Whole-Brain 3D Myeloarchitectonic Atlas: Mapping the Vogt-Vogt Legacy to the Cortical Surface, Neuroimage Volume 263, November 2022, 119617 10.1016/j.neuroimage.2022.119617

## Value of the Data

• This 3D myeloarchitectonic atlas builds on both meta-analyses-derived and ground-truth histological data, providing access to qualitative and quantitative information on cortical myeloarchitectonics.
• Since MRI surrogate markers of myelin demonstrate close overlap with histological cortical parcellations, this atlas can be utilized to further explore biological validity and underpinnings of novel, non-invasive metrics or sequences in pathological conditions
• Due to its seamless integration with widely used neuroimaging analysis software such as FreeSurfer, MYATLAS can inform researchers and clinicians studying *in vivo* cortical myelin in health and disease. To facilitate its use, the atlas has been expanded to several commonly used brain templates.

## 1 Objective

Myeloarchitectonics, i.e. the parcellation of the cortex into distinct areas according to layering, arrangement, packing and density of myelinated fibers and bundles, has been the focus of Oskar Vogt and associates at the beginning of the 20th century [2]. Nieuwenhuys and co-workers mapped this legacy histology data to a non-digital, 2D representation of the Montreal Neurological Institute (MNI) Colin27 brain template. While such maps facilitate access to historical postmortem data, contemporary neuroimaging analyses require 3D stereotaxic representations. We constructed a 3D, whole-brain stereotaxic myeloarchitectonic atlas in common space by harnessing manual translations of the Vogt-Vogt atlas to the Colin27 brain template [1].

Here we share a) ready-to-use myeloarchitectonic parcellations in common space aligned with several brain templates in both volumetric and surface formats, b) raw and processed histology slices used for constructing the atlas and their original publications, c) intracortical laminar profiles of myelin content derived from photometric data of Vogt and Hopf, and, finally, d) Bash shell and MATLAB scripts to apply myeloarchitectonic parcellations to new projects.

## 2 Data Description

### 2.1 Scene Files in wb_view Format

“Scene files” for the connectome workbench “wb_view” facilitate data handling and visualization [3]. Six scene files are included in this dataset, which were created to summarize secondary data and facilitate use with modern neuroimaging toolkits such as FreeSurfer and the human connectome environment. All .scene files are located in \maps\Surface\ subdirectory of the GitHub node,

Colin27.scene illustrates the newly developed myeloarchitectural atlas, superimposed onto the MNI-Colin27 brain template in MNI space, as well as accompanying maps of cortical myelin density (MGL). Index2 of this scene file contains maps of fiber bundle intrusion and fiber orientation types per cortical field as well as general myeloarchitectonic categories introduced by Vogt.

MRI-Histology.scene contains in-vivo and histological validation maps. Index1 illustrates *in vivo* T1q data myelin proxy data cross-correlated to histology-derived MGL. Index 2 and 3 contain cytoarchitectonic feature maps (gyral thickness and cell size), correlated with MGL of MYATLAS. conte69_10k.scene and conte69_32k.scene contain MYATLAS cortical segmentations for use with FreeSurfer Conte69 brain templates, matched to the respective vertex resolution, i.e*.* 10 and 32 k vertices. For Conte69, inflated surface maps are also included (black and white). fsaverage.scene and fsaverage5_scene contains MYATLAS cortical segmentation for use with FreeSurfer fsaverage brain templates in their native space, for both FreeSurfer 5 and 6 releases.

### 2.2 Surface-Based Atlas Representations

These files contain individual myeloarchitectonic cortical areas created by manual cortical delineation directly on the pial surface of MNI-Colin27 in MNI space (GitHub directory /maps/Surface/Freesurfer_COLIN27/annot/). All cortical areas in *.label format are given as single annotation files for the left (lh.*) and right (rh.*) hemisphere. For reference purposes, all individual Vogt areas in *.label file format are also available. rh.colin27.vogt_vogt.annot contains Vogt-Vogt parcellations for the right hemisphere based on the MNI-colin27 brain template in MNI space lh.colin27.vogt_vogt.annot contains Vogt-Vogt parcellations for the right hemisphere based on the MNI-colin27 brain template in MNI space rh.colin27.Baillarger_type.annot contains a map of fiber bundle types, i.e. myeloarchitectonic categories according to the behavior of the bands of Baillarger per each Vogt cortical area, superimposed on to the MNI-colin27 brain template in MNI space rh.colin27.Intrusion_type.annot complements the myeloarchitectonic parcellations by describing Vogt areas according to their bundle intrusion type, i.e. penetration patterns of tangential and radial fiber bundles

### 2.3 Volumetric Atlas Representations

Individual cortical areas in FreeSurfer label format were converted into volumetric *.nifti files and combined into a single hemispherical volume atlas, aligned with the MNI-Colin27 brain template in common space. Separate files for each hemisphere (_rh for the right and _lh for the left hemisphere) as well as a whole-brain atlas with a corresponding reference table are provided. For reference purposes, all individual cortical areas in volumetric nifti file format are also available in a *.tar archive. Both hemispheric, whole brain and individual cortical files are available from /main/maps/Volume/ on GitHub. vogt_multilabel_rh.nii and vogt_multilabel_lh.nii These volumetric atlas files in MNI space contain all Vogt areas of the individual right and left hemisphere, aligned with the MNI ICBM152 brain template, while vogt_bilateral_myatlas.nii contains labels for both hemispheres. These files are best used together with the accompanying look-up table label-descriptions_vogt-labels.txt for referencing and label color assignment. This file can be used in volumetric viewers such as ITK-Snap or mricron label-descriptions_vogt-labels.txt The reference table for volumetric atlas files contains label numbers and names as well as color codes per each cortical area vogt-labels_nifti.tar contains individual Vogt labels for both hemispheres as a reference. These areas were used to create the individual and bilateral atlas map as described above

### 2.4 Myeloarchitectural Reference and Lookup Tables

These Excel files contain information on fiber bundle penetration patterns, myelin density and light absorption profiles (i.e., quantitative myelin contents in a machine-readable and clear fashion). The txt reference tables contain further myeloarchitectonic information per each cortical field, i.e. Baillarger band type, bundle intrusion type and original Vogt label names with RGB colorization information for use with volumetric visualization software (.txt) and FreeSurfer (.ctab). All lookup-tables and other files mentioned here are located in the /main/ GitHub directory, titled accordingly and include: myeloarchitectectonic_table.xlsx This file contains label descriptions, area numbers, myeloarchitectonic characteristics such as Baillarger bands and bundle intrusion types and their respective source publication. It is intended for use as a machine-readable source of data, e.g. for reference purposes as a Matlab variable. myeloarchitectonic_table_clear.xlsx represents the human-readable version and is intended as a quick-reference for myeloarchitectural properties of a given brain area label_list_Baillarger_type.txt, label_list_Intrusion_type.txt and label_list_vogt_vogt.txt: These files contain information on the Baillarger band and bundle intrusion types per each cortical field and are intended for use with volumetric visualization toolkits

Intrusion_type.ctab, vogt_vogt.ctab, vogt_vogt_new.ctab. These ctab files are intended as reference files for FreeSurfer. Intrusion_type.ctab contains the same myeloarchitectural categories and -properties per each individual field like the txt files. Both vogt_vogt.ctab and vogt_vogt_new.ctab are color maps and contain color references for the surface-based atlas files. The “new.ctab” file represents the colorization of the scene files, with colors assigned to areas according to their myeloarchitectural properties.

### 2.5 Shell Scripts

Two shell scripts for applying MYATLAS to newly acquired *in vivo* subject data for use with FreeSurfer are provided. mapping_colin27_labels_onto_individuals.sh and mapping_colin27_labels_onto_individuals_batch.sh are used for newly acquired data. The file depth_profiling_generic.py *ca*, a Python script for newly acquired histology data is available for image normalization and depth-profiling as described in the methods section of the original publication. These shell scripts are available from the /main directory.

### 2.6 Histology_raw.zip

This file contains raw and normalized Vogt myeloarchitectonic histology slices together with their corresponding cortical depth profile. All files are labelled according to their cortical field. The zip archive is located in the /main/ directory.

## 3 Experimental Design, Materials and Methods

### 3.1 Manual Cortical Labeling and 3D Myeloarchitectonic Atlas Creation

All original 2D illustrations by Nieuwenhuys [4] were split into eight separate view planes (lateral, medial, superior, inferior, orbitofrontal, supratemporal, parietal opercular, insular), in accordance with previous procedures [5]. These view planes allow for detailed and multi-angle structure tracing. A single rater manually labeled all cortical areas by cross-referencing geometric landmarks between the original 2D illustration and the convexity of the 3D Colin27 brain template surface using FreeSurfers visualization and mapping tool “*tksurfer*” (surfer.nmr.mgh.harvard.edu/fswiki/TkSurfer). To facilitate cross-referencing, prominent sulci and gyri on the original 2D Colin27 map such as the frontoparietal sulcus were used as systematic landmarks, since these are easily recognizable in a 3D representation. In order to reach optimal anatomical matching of area boundaries, we further relied on the main gyro-sulcal patterns surrounding each region. Accuracy of each cortical label was then verified by a second rater. For area mismatch across view planes or between 2D illustrations and 3D cortical surface, an inter-rater consensus was reached to minimize discrepancies and manual corrections were applied when necessary. All manually segmented labels were numbered according to Vogt numeric conventions and merged with color tables to create a single annotation file, containing 214 parcellations (Fig. 1C of the research article). Finally, all parcellations and gray-level intensity maps are available in gifti and nifti [‘*dscalar* for mean gray levels (MGL) and ‘*dlabel* for parcellation] format, aligned with three brain spaces (MNI-Colin27, Conte69 and FSAVERAGE).

### 3.2 Quantitative Myelin Content and Cortical Depth Profiling

First, histologically-stained microphotographs available from the original publications [6], [7], [8], [9], [10], [11], [12] were screen-captured with a fixed format and size, followed by estimating gray level intensity (i.e., a surrogate of light absorption across cortical laminae to quantify cortical myelin density). The digitized histological figures were normalized to allow for comparisons with the python script described in the data section. Normalized intensity values were then plotted as a depth profile, representing myelin density across the full cortical thickness. Other information on myeloarchitectonic features, such as fiber bundle types and layer-specific density of each slice were also extracted. These architectural features were then compiled into lookup-tables and mapped onto MYATLAS surfaces, creating feature-weighted category maps (Fig. 2 in the original work).

### 3.3 Cross-correlating *In Vivo* Myelin Imaging and Myeloarchitectonic Features

As a second step, we examined reliability of *in vivo* T1q-weighted MRI, serving as a myelin surrogate marker, against the legacy ground-truth histology data. For this, we randomly selected quantitative T1w data of 202 healthy individuals from the Leipzig Study for Mind-Body-Emotion Interactions (LEMON) dataset [13]. In FreeSurfer, we positioned 10 equivolume surfaces between the inner and outer cortical interface using the function *equivolumetric_surfaces.py* (https://github.com/kwagstyl/surface_tools.git). These surfaces systematically sampled an axis perpendicular to the cortical ribbon, with interpolation at each vertex [14]. Following raw value extraction, we correlated *in vivo* data with depth profiles acquired from the digitized histology slices.

### 3.4 Relationship with Cytoarchitectonic Features

To further explore the neurobiological validity of MYATLAS, we investigated relationships of myelin density, as represented by MGL, with cytoarchitectonic features, including gyral dome thickness, cellular density and cell size [15]. Since gyral dome thickness is usually given as a range, averages per cortical area were calculated. Cellular density was averaged across cortical laminae, whereas cell size was indexed according to [Hmean x Wmean]; with Hmean (Height) = [H(min-max)/2] and Wmean (Width) = [W(min-max)/2] per individual lamina and then equally averaged. To facilitate between-atlas comparisons, all area boundaries were matched using a winner-takes-all approach, thus assigning each parcel of the von Economo-Koskinas’ atlas to our cortical segmentation.

## Ethics Statements

Institutional Review Board approval was waived due to the publicly available character of data. Information on ethics board review approval of the LEMON dataset has been previously published [13]. All procedures followed were in accordance with institutional guidelines and the Declaration of Helsinki.

## CRediT authorship contribution statement

**Niels Alexander Foit:** Data curation, Methodology, Formal analysis, Conceptualization, Writing – review & editing. **Seles Yung:** Software, Formal analysis. **Hyo Min Lee:** Writing – review & editing. **Andrea Bernasconi:** Supervision, Conceptualization, Writing – review & editing. **Neda Bernasconi:** Supervision, Conceptualization, Writing – review & editing. **Seok-Jun Hong:** Data curation, Supervision, Methodology, Formal analysis, Writing – review & editing.

## Declaration of Competing Interest

The authors declare that they have no known competing financial interests or personal relationships that could have appeared to influence the work reported in this paper.

## Data Availability

Myatlas - Whole-Brain myeloarchitectonic histology atlas (Original data) (BALSA).
